# Campaigning for a fact-based approach to health journalism

**DOI:** 10.2471/BLT.17.030417

**Published:** 2017-04-01

## Abstract

Gary Schwitzer argues that – when it comes to reporting on health and medicine – the news media in the United States of America are often out of touch with the public they purport to serve. He talks to Fiona Fleck.

Q: How did you become interested in public health?

A: As a newsroom reporter 25 or more years ago, I remember realizing that I didn’t want to follow blindly what the other journalists were doing. I didn’t want to simply report on claims about new treatments, tests, products and procedures. It’s easy for us, journalists, to congratulate ourselves because we’ve met the day’s quota of news, but too often we accept the messages that are spoon fed to us by the pharmaceutical industry and others with vested interests rather than digging more to find out what is really going on. I wanted to concentrate on more important aspects of health, health care and health policy than we were covering in the news business.

Q: Why do journalists accept this situation?

A: Journalists have less time to churn out their news stories and easily fall prey to the messages of vested interests in public relations (PR) news releases. Journalists believe that they must often match what the competition is reporting. They don’t always have time to do the background research and check the facts. Sometimes they don’t have the knowledge or skills to critically evaluate PR news releases. They are under so much pressure to get the story out that balanced reporting sometimes falls by the wayside. There is fierce competition among news organizations and so journalists think that their reports need to be sensational to get the attention.

Q: “Post-factual” and “alternative facts” have become fashionable expressions for publicising incorrect and unreliable information, but is this really new in health-care reporting?

A: During the course of my career, we saw a big change in health-care reporting in the mid-1980s in the United States – possibly in other countries too – especially in the news coverage of what I call the three As: AIDS, Alzheimer disease and the artificial heart. It was easy to be swept along by that. Suddenly bold claims of dramatic breakthroughs were appearing on front pages, in magazine cover stories and on network television news. When I left journalism in 1990, I joined researchers at Dartmouth Medical School interviewing newly diagnosed patients about their treatment decision-making dilemmas and, clearly, the media were not helping them. In journalism school, we are taught that you must have your audience in mind and that you should think of real people, otherwise you will not be an effective communicator. Health-care journalism does not always reflect this. Instead, we are promoting the flashy, sexy and dramatic without helping people develop an understanding of health issues.

Q: Why?

A: In some parts of the world the news economy is suffering from cutbacks of staff. Fewer journalists are expected to do more with less. Copy-editing and research positions have been cut, which reduces the quality of the news. Reporters are expected to file news stories in different formats: for the web first, then the print edition – if there still is one – then digital photos as well as audio, video and social media. Since the digital revolution in the early 2000s, print circulation of newspapers has been declining in the United States. News organizations concentrate on their online presence and are driven by click rates. Academic institutions and journals are also competing for media coverage and sensationalizing their news releases. In all of this, we lose sight of readers’ interests.

Q: Since the online media revolution, readers have been able to discuss news articles online. Doesn’t this make journalists more responsive to readers’ needs and more accountable?

A: It would make journalists more responsive, if they actually engaged with the readers in these online forums. Many journalists I know abhor online comment sections. Earlier this year I wrote about a *New York Times* story that reported “Pregnant women may want to avoid licorice, which may affect the cognitive abilities of their children.” Some readers left comments online, calling it “bad science reporting,” reminding the paper that “association is not cause and effect,” and “correlation is not causation.” That is a sign of the wisdom of the crowds. I wrote, “Smart readers are catching on, tired of the flood of fearful or fanciful health-care news. You’d better listen to your readers. You’re losing them. And this is one reason why.”

Q: Tell us about HealthNewsReview.com and why you set it up?

A: The pioneers of this movement were a group of researchers at the University of Newcastle in Australia. They set up Media Doctor in 2004, a website dedicated to improving the accuracy of health reporting, which inspired initiatives in Canada, Germany, China (Hong Kong Special Administrative Region), Japan and ours in the United States. We hoped that together we could pool our data and hold up a clear data-driven mirror to editorial decision-makers around the globe. Unfortunately that never happened. Today, only our operation and the one in Germany are still running. We adopted the original 10 criteria established by the Australian team for assessing the quality of reporting on health and medical care, and we still use them. HealthNewsReview.org became the biggest and most active initiative in terms of output and reach. This year we celebrate our 11th anniversary. We have three full-time and two part-time editorial staff. To date, we have reviewed more than 2300 news stories and more than 330 PR news releases.

“We hoped that together we could pool our data and hold up a clear data-driven mirror to editorial decision-makers around the globe.”

Q: How do you assess and rate articles?

A: We have about 50 external reviewers. Three reviewers assess each article, applying our 10 criteria. These include whether the journalists have adequately considered the cost of the intervention, its potential harms and benefits, whether they had compared new ideas with existing alternatives, and whether they solely relied on a press release or used independent sources. About half of the reviewers have medical degrees, a PhD or other advanced degrees and are specialized in the evaluation of evidence. About half of our reviewers are also journalists or academic institution-based science writers or science communications specialists. We also have a few patients who serve as reviewers. 

Q: How well do the news reports and PR releases do? 

A: When journalists or PR news release writers are reporting on new drugs or other health-care interventions, they tend to make them look terrific, risk free and without a price tag. Most health-care news releases and stories do not promote an informed health-care consumer population. It’s a missed opportunity. The potential of the mass media to do good is great: that’s why I went into journalism and why I’ve remained dedicated to health-care journalism. 

Q: What are the consequences of inaccurate reporting in the media of health and medical stories?

A: We whip the “the worried well” into a frenzy. We drive people to see their physicians, and promote undue demand for unproven interventions. We disease-monger, medicalizing normal states of health, and make healthy people seek unnecessary treatment. When I give talks to physician audiences, they often tell me how much time they spend debunking claims that their patients have read in the media. We are obsessed with numbers that have no relevance for our health. It bothers me as a life-long journalist when I see that we are steering people in the wrong direction. Inaccurate, imbalanced, incomplete media messages about health care are doing harm to people.

Q: How are these messages harming people?

A: At HealthNewsReview.org we produce audio podcasts of people describing how they were harmed by misleading media messages. A man with a brain tumour was misled by a company news release that made claims about a breakthrough drug that were not properly filtered by the news media. His hopes for treatment were raised, only to be crushed when his doctor told him the drug was not ready for human use. Two women with breast cancer told us how difficult it is to make treatment decisions when there are conflicting news stories, or when celebrities make news about their own treatment decisions that may have no relevance to other women. We know that editorial decision-makers don’t set out to hurt people. But the harms we point to, though unintentional, are nonetheless real.

Q: Apart from monitoring the news media and PR releases, how can media coverage of health-care news be improved? 

A: Some health, medical and science journalism meets a high standard. Some of these are foundation-supported efforts in collaboration with major news organizations. Philanthropic foundations in the USA are playing an important role in helping journalism through difficult times. But the troughs between those peaks of excellence are becoming deeper and wider. For example, some news media mistakenly promote animal research and early phase I drug trials, as if the products were available at the corner drug stores. Others are claiming falsely that observational research can prove cause and effect. We developed a primer for journalists and the public on how to interpret observational data and other technical aspects of health-care reporting. Our message is that if it is too technical for journalists it’s better not to report, than to report something that is not accurate. And despite the intense competition, it’s better to be second and correct than to rush to be first and be wrong.

“It’s better not to report, than to report something that is not accurate.”

Q: What are your hopes for the future of health-care news reporting?

A: There should be room for promoting health literacy, for example, explaining that people should focus on *absolute* not *relative* risk reduction. They should not be amazed by claims that a drug reduced the risk of a problem by 50% (relative risk reduction) when that may mean that the absolute risk reduction was only from 2 in 100 in the untreated group to 1 in 100 in the treated group – a 1% absolute risk reduction. We can do far more good with our media messages if we explored the social determinants of health, how to judge medical evidence and how to evaluate the quality of health care. Issues of access to care and disparities in care don’t get enough attention. We can do a better job.

